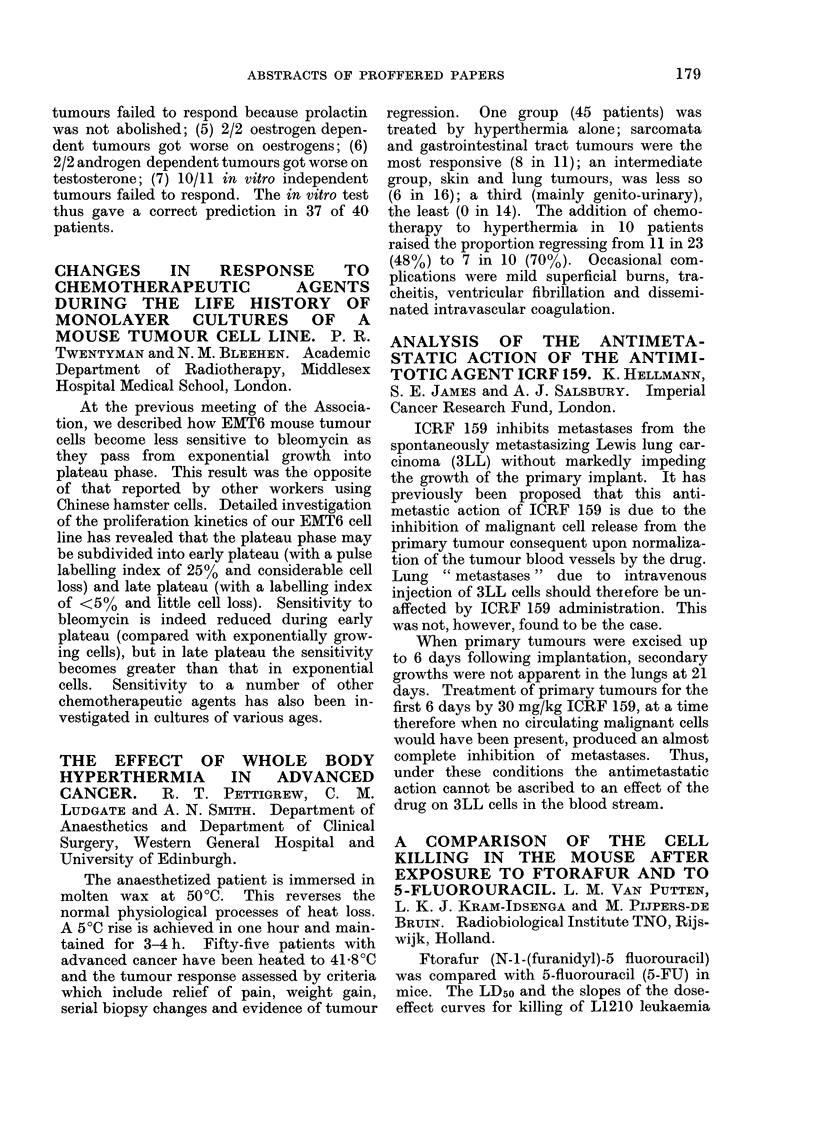# Proceedings: Changes in response to chemotherapeutic agents during ther life history of monolayer cultures of a mouse tumour cell line.

**DOI:** 10.1038/bjc.1974.151

**Published:** 1974-08

**Authors:** P. R. Twentyman, N. M. Bleehen


**CHANGES IN RESPONSE TO CHEMOTHERAPEUTIC AGENTS DURING THE LIFE HISTORY OF MONOLAYER CULTURES OF A MOUSE TUMOUR CELL LINE.** P. R. Twentyman and N. M. Bleehen. Academic Department of Radiotherapy, Middlesex Hospital Medical School, London.

At the previous meeting of the Association, we described how EMT6 mouse tumour cells become less sensitive to bleomycin as they pass from exponential growth into plateau phase. This result was the opposite of that reported by other workers using Chinese hamster cells. Detailed investigation of the proliferation kinetics of our EMT6 cell line has revealed that the plateau phase may be subdivided into early plateau (with a pulse labelling index of 25% and considerable cell loss) and late plateau (with a labelling index of <5% and little cell loss). Sensitivity to bleomycin is indeed reduced during early plateau (compared with exponentially growing cells), but in late plateau the sensitivity becomes greater than that in exponential cells. Sensitivity to a number of other chemotherapeutic agents has also been investigated in cultures of various ages.